# Enhancing protein production and growth in chinese hamster ovary cells through miR-107 overexpression

**DOI:** 10.1186/s13568-024-01670-y

**Published:** 2024-02-01

**Authors:** Maryam Jari, Shahriyar Abdoli, Zahra Bazi, Fatemeh Tash Shamsabadi, Farnaz Roshanmehr, Majid Shahbazi

**Affiliations:** 1 Medical Cellular and Molecular Research Center, Golestan University of Medical Sciences, Shastkola Road, Falsafi Complex, Gorgan, Zip code: 4934174611 Iran; 2 Department of Medical Biotechnology School of Advanced Technologies in Medicine, Golestan University of Medical Sciences, Gorgan, Iran; 3 AryaTina Gene (ATG) Biopharmaceutical Company Gorgan, Gorgan, Iran

**Keywords:** Erythropoietin (EPO), miR-107, microRNA, Protein Production, Cell Growth, Chinese hamster ovary cells

## Abstract

Chinese Hamster Ovary (CHO) cells are widely employed as host cells for biopharmaceutical production. The manufacturing of biopharmaceuticals poses several challenges, including restricted growth potential and inadequate productivity of the host cells. MicroRNAs play a crucial role in regulating gene expression and are considered highly promising tools for cell engineering to enhance protein production. Our study aimed to evaluate the effects of miR-107, which is recognized as an onco-miR, on erythropoietin-producing CHO cells (CHO-hEPO). To assess the impact of miR-107 on CHO cells, a DNA plasmid containing miR-107 was introduced to CHO-hEPO cells through transfection. Cell proliferation and viability were assessed using the trypan blue dye exclusion method. Cell cycle analysis was conducted by utilizing propidium iodide (PI) staining. The quantification of EPO was determined using an immunoassay test. Moreover, the impact of miR-107 on the expression of downstream target genes was evaluated using qRT-PCR. Our findings highlight and underscore the substantial impact of transient miR-107 overexpression, which led to a remarkable 2.7-fold increase in EPO titers and a significant 1.6-fold increase in the specific productivity of CHO cells (p < 0.01). Furthermore, this intervention resulted in significant enhancements in cell viability and growth rate (p < 0.05). Intriguingly, the overexpression of miR‑107 was linked to the downregulation of *LATS2, PTEN,* and *TSC1* genes while concurrently driving upregulation in transcript levels of *MYC, YAP, mTOR,* and *S6K* genes within transgenic CHO cells. In conclusion, this study collectively underscores the feasibility of utilizing cancer-associated miRNAs as a powerful tool for CHO cell engineering. However, more in-depth exploration is warranted to unravel the precise molecular intricacies of miR-107's effects in the context of CHO cells.

**Supplementary Information:**

The online version contains supplementary material available at 10.1186/s13568-024-01670-y.

## Introduction

As a result of the increasing market demand for biological products, biopharmaceutical companies are compelled to undertake expansion initiatives to accommodate and meet the growing needs of the industry. Consequently, the imperative need to enhance the efficiency of cell lines utilized as hosts for the production of recombinant proteins becomes increasingly significant (Mullard 2021; Szkodny and Lee 2022). CHO cells are widely used for generating complex, glycosylated, and challenging-to-produce proteins (Keysberg et al. 2021; Lalonde and Durocher 2017).

CHO cells produce 70% of biopharmaceuticals and nearly all mAbs (Lalonde and Durocher 2017). Their widespread usage is attributed to several factors, including high productivity, consistent growth characteristics, suitability for large-scale industrial cultivation, adaptability to diverse chemically defined media, reduced susceptibility to human viral infections, and the ability to perform human-compatible glycosylation (Dahodwala and Lee 2019; Kim et al. 2012; Kunert and Reinhart 2016; Lai et al. 2013; Tihanyi and Nyitray 2020). The unveiling of the Chinese Hamster and CHO-K1 genomes has significantly facilitated the targeted genetic engineering of CHO cells (Brinkrolf et al. 2013; Lewis et al. 2013; Xu et al. 2011).

Despite the widespread use of CHO cell platforms for producing recombinant proteins, inherent limitations remain in the production and secretion of many complex proteins. These limitations encompass low productivity, growth impediments, expression instability, inadequate resistance to culture-related stresses, and excessive production costs (Kaneyoshi et al. 2019). Hence, diverse endeavors have aimed to enhance the performance of CHO-producing cell lines to heighten productivity. The predominant strategy for host cell engineering has involved the overexpression of genes that promote cell proliferation, longevity, stress resistance, apoptosis evasion, protein production, and secretion. Notably, the overexpression of essential genes involved in cell metabolism, protein biosynthesis, and glycosylation has been proven to be effective in augmenting growth, productivity, and product quality superiority (Fischer et al. 2015a, b; Keysberg et al. 2021; O’Flaherty et al. 2020; Tihanyi and Nyitray 2020).

A promising new cell engineering strategy gaining traction is the manipulation of micro-RNA (miRNA)(Huhn et al. 2019). The endogenous, highly conserved short non-coding RNAs, known as miRNAs, regulate gene expression within eukaryotic cells (Berezikov 2011). With their compact 7–8 nucleotide recognition sequences, miRNAs can post-transcriptionally modulate the expression of numerous protein-coding genes or entire pathways (Bartel 2009; Hackl et al. 2011; Tihanyi and Nyitray 2020). A single miRNA can regulate multiple mRNA targets without straining the cell's translational machinery. This unique property has sparked growing interest within the biopharmaceutical industry, highlighting microRNAs as potent tools for cell engineering (Jadhav et al. 2013; Valdés‐Bango Curell and Barron 2018). Therefore, the miRNA regulatory system is a practical molecular approach for orchestrating entire cell signaling pathways. Despite the progress, further investigations remain imperative to unravel the full extent of miRNA effects and mechanisms on recombinant protein expression in CHO cells (Liu et al. 2022).

Several studies have used miRNAs to enhance cellular productivity in CHO cells based on previous understanding of the capabilities of miRNAs (Bazaz et al. 2023; Kelly et al. 2015; Singh et al. 2022). The optimization of the CHO cell line for enhanced protein production has been effectively achieved through miRNA overexpression (e.g., miR-23, miR-2861, miR-17) or downregulation (e.g., miR-7, miR-14, miR-106b) (Coleman et al. 2019; Fischer et al. 2015a, b; Jadhav et al. 2014; Kelly et al. 2015; Xu et al. 2019a). A recent study utilized the CRISPR/Cas9 genome editing system to delete miR-744, aiming to enhance CHO bioprocess performance (Raab et al. 2019). Moreover, manipulation of miRNA-mediated regulation has been applied to amplify the production of challenging-to-express novel biotherapeutics (Schoellhorn et al. 2017; Tharmalingam et al. 2018). Notably, a recent investigation successfully increased cell growth, survival, and productivity by overexpressing miR-32 in CHO cells (Bazaz et al. 2023).

Various strategies have been employed to identify miRNAs as a target for cell engineering. A prominent approach involves screening methods to select miRNAs with the potential to enhance cell productivity using previously identified miRNAs in human cancer research, which have been found to influence cell growth properties (Inwood et al. 2018). Dysregulated miRNAs that modulate cell growth have been extensively documented in human cancers, acting either as proliferation promoters or cell death signal inhibitors (Peng and Croce 2016). Specific miRNAs have been assessed for their capacity to enhance recombinant protein expression (Inwood et al. 2018). Additionally, contemporary bioinformatics evaluations offer precise avenues for uncovering miRNA targets with greater accuracy.

In this study, we sought to leverage the potential of miRNAs derived from cancer-associated upregulated miRNAs to enhance the productivity of CHO cells. By considering miR-107’s regulatory function in human cancer and its conserved sequence between humans and hamster genomes (Crisetulus griseus), coupled with an exploration of both predicted and validated target genes of miR-107 and its effects on cell proliferation, growth, and viability, we hypothesized that the overexpression of miR-107 in our CHO-hEPO cells could yield elevated viable cell density and enhances protein production.

In this research, our focus centers on evaluating the effects of transient miR-107 overexpression on crucial aspects of CHO-K1 cell behavior. Specifically, we investigate its influence on cellular growth, viability, and productivity within a CHO-K1 cell line engineered for stable recombinant Erythropoietin (EPO) expression. EPO, a naturally occurring glycoprotein hormone with a molecular mass of 30.4 kDa, is synthesized by renal peritubular cells and primarily stimulates red blood cell production (Jelkmann 2013, 2016).

Additionally, conducting a comprehensive literature review and employing in silico analysis has determined that miR-107 affects cell growth and protein synthesis by targeting specific genes. Among the identified target genes, we examined the expression levels of *LATS2* and *PTEN*, along with their respective cellular pathways. The YAP and PI3K signaling pathways facilitate cellular growth and protein synthesis. The genes whose expression levels were measured by qPCR included *PTEN, TSC1, LATS2, MTOR1, S6K, YAP, and MYC*.

Our study investigates the potential of miR-107 as a modulator of these cellular attributes, providing a foundation for understanding its role in optimizing biopharmaceutical production.

## Material and methods

### Cell lines and cell culture

The CHO-K1 cell line was donated by the Pasture Institute (Tehran, Iran). CHO cells producing human Erythropoietin (CHO-hEPO) were prepared in our laboratory at Golestan University of Medical Sciences as part of a project funded by grant number 111167. This cell line was derived from a CHO-K1 cell line transfected with a plasmid containing the human EPO gene (accession number M11319) and the puromycin resistance gene. The generation of the stable CHO cell line producing hEPO was achieved by subjecting the cells to selective pressure using 5 μg/ml puromycin. The cell lines were cultured in DMEM-F12 media (Gibco, Life Technologies Inc., New York, USA), supplemented with 1% (v/v) penicillin/streptomycin (Sigma–Aldrich, MO), and 10% (v/v) fetal bovine serum (Gibco, Life Technologies Inc., New York, USA), at 37 ºC in a humidified atmosphere containing 5% CO_2_.

### MicroRNA selection

The process of miRNA selection for potential CHO cell engineering candidates began with a comprehensive review of pertinent studies focusing on upregulated miRNAs implicated as regulators of cell proliferation in human cancer research. Furthermore, to ensure cross-species applicability, we specifically targeted miRNAs with conserved sequences in humans and hamsters (*Crisetulus griseus*) (Additional file 2: Figure S1). Subsequently, candidate target mRNAs with potential binding sites for individual miRNAs were pinpointed through a systematic search across publicly available databases enriched with prediction algorithms. Notably, these databases included RNA22, Targetscan, miRWalk, and miRanda, which can be accessed respectively at https://cm.jefferson.edu/rna22/, http://genes.mit.edu/targetscan/, http://mirwalk.umm.uni-heidelberg.de, and http://www.microrna.org/mammalian/index.html). To enhance the analytical rigor, our study exclusively considered target genes that emerged as hits across all four algorithms. Also, extended scrutiny was performed to find validated target genes from the scientific literature and the miRTarBase database (Additional file 1). The selected microRNA candidate, cgr-miR-107 (MIMAT0023734; AGCAGCAUUGUACAGGGCUAUC), was sourced from the comprehensive miRBase repository (www.mirbase.org).

### Plasmid construction and transfection

**Fig. 1:**
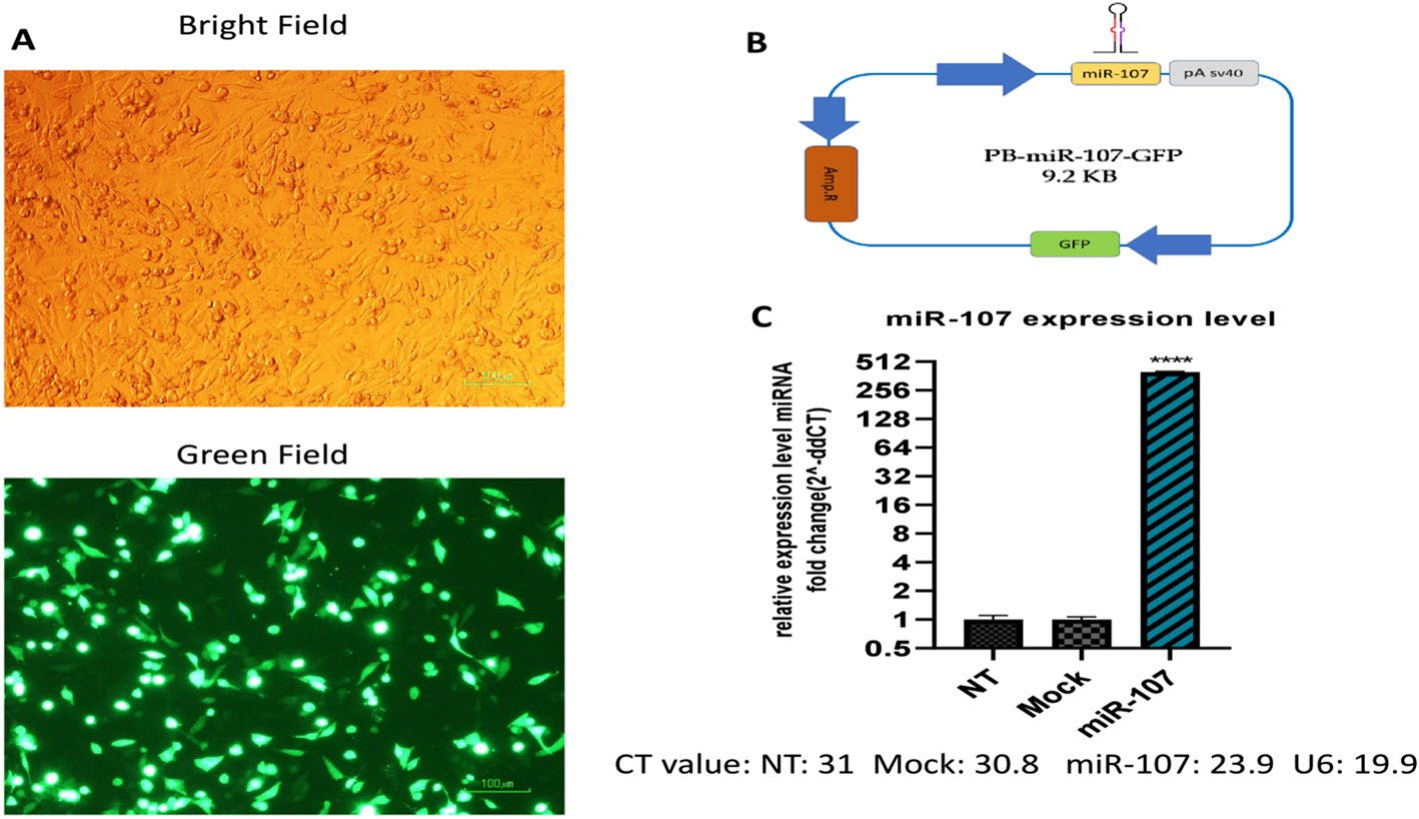
Overexpression of miR-107 in CHO Cells. **A** Fluorescence microscopy image displaying successful transfection. **B** Schematic view of the PB-miR-107-GFP vector. **C** Comparative analysis of miR-107 expression levels using qRT-PCR in miR-107 transfected cells, Mock and non-transfected (NT) cells. The miRNA expression is illustrated as fold-change relative to the controls and normalized to U6 snoRNA. The statistical significance of differences between miR-107 transfected cells and controls was determined (**** *p* < 0.0001)

To achieve the overexpression of miR-107 in CHO-hEPO cells, a cassette encompassing the sequence of miR-107 was synthesized (Gene Scripts, China) (see Additional file 2: Figure S2). This sequence was cloned into the PB513b-1 Vector system (SBI, USA) between the *Spe*I and *Eco*RI sites. The resulting construct, the PB-miR-107-GFP vector, was utilized for subsequent transfection procedures (Fig. 1B). In parallel, the PB513-b1 vector was also transfected into a distinct cell group and was used as a Mock control.

Briefly, 2 × 10^5^ viable CHO- hEPO cells in the exponential growth phase were seeded into a 6-well plate. Subsequently, they were transfected with the desired plasmid using ScreenFect®A according to the manufacturer's protocol. Non-transfected CHO-hEPO cells (NT) were incorporated as an additional control group for rigorous comparison. This enabled a comprehensive examination of productivity, growth, and viability, thereby enhancing the accuracy of the experimental results.

### RNA extraction and quantification of mRNA and miRNA levels

Total RNA extraction from three distinct cell groups, miR-107, NT, and Mock, was performed using TRIzol® reagent (Biobasic, Canada) per the manufacturer’s protocol. RNA quality was quantified and assessed using the Picodrop Microliter UV/Vis Spectrophotometer Model PICOPET01 (Picodrop, UK).

Total RNA, comprising 1 μg, underwent reverse transcription using the BONmiR stem High Sensitivity MicroRNA 1st Strand cDNA Synthesis kit (BonYakhte, Iran) following the manufacturer’s protocol. Specific stem loops were utilized in the reverse transcription process to capture miRNAs.

Quantitative PCR (qPCR) for miRNA involved the utilization of specific miR-107 forward and universal reverse primers in conjunction with the SYBR green master mix (Ampliqon, Denmark). The U6 snRNA gene served as the reference gene. The reactions were conducted on the StepOne real-time PCR System (Applied Biosystems, USA).

For assessing mRNA levels, reverse transcription was executed using the Yekta Taihiz Azma cDNA Synthesis Kit (YT4500; Tehran, Iran). The beta-actin gene was used as an internal control for mRNA quantification.

Notably, each experiment was accurately conducted in triplicate to ensure precision and reliability. The delta-delta Ct method was employed to determine the fold change (FC) between the control and treatment groups. Graphical representation of data includes error bars depicting standard deviation (SD). The list of primers and stem-loop sequences used is provided in Table S1.

### Cell cycle analysis

To assess the impact of miR-107 overexpression on cell cycle progression, CHO-hEPO-miR-107, Mock, and non-transfected CHO-hEPO (NT) cells were subjected to propidium iodide (PI) staining (BioLegend, England). Initially, 1 × 10^6^ cells per group were counted, washed with PBS, and fixed with 70% ethanol. The fixed cells were resuspended in PBS containing 100 µg/ml RNase A and incubated at 37 °C for 30 min. Finally, the cells were stained with 50 µg/ml PI in a dark environment for 15 min. The cell cycle distribution was evaluated using a BD Accuri C6 flow cytometer. Pulse processing was used to exclude cell doublets. This can be achieved by using the pulse area versus pulse width. Subsequently, it was applied to the PI histogram plot. The percentage of cells in each phase of the cell cycle was quantified using ModFit software (BD Biosciences).

### EPO quantification via immunoassay

To evaluate the impact of miR-107 overexpression on productivity, 2 × 10^5^ CHO-hEPO cells were transfected with the PB-miR-107-GFP vector (referred to as the miR-107 group) and with pb531-b1 (serving as a Mock) in a 6-well plate. Non-transfected CHO-hEPO cells (NT) were used as an additional control group. The cell culture supernatant from each group (miR-107, Mock, and NT) was collected, and the concentration of secreted EPO was measured.

EPO concentration was quantified using the Immulite EPN kit (L2KEPN2) produced by Siemens (Llanberis, Gwynedd, UK). The analysis was facilitated using the IMMULITE 2000 XPi immunoassay system, an automated chemiluminescent immunoassay analyzer, following the protocols outlined by the manufacturer (Siemens Healthineers, Germany). The specific productivity (pg/cell/day) was derived through the following Eq. 1, where CP (μg/ ml) represents the EPO concentration, and VCC (cells/mL) denotes the viable cell concentration (Maccani et al. 2014).

1 $${{\text{q}}}_{p}=\frac{CP}{{\text{VCC}}}\times {10}^{6}.$$

### Evaluation of cellular growth and viability

To assess the effect of miR-107 overexpression on growth rate and viability, 5 × 10^4^ CHO-hEPO cells were seeded in a 24-well plate. Twenty hours after seeding, the cells were transfected with the desired vectors. The viable cell density (VCD) and their overall viability in each group (miR-107, Mock, and NT) were assessed 24, 48, and 72 h after transfection using the trypan blue dye exclusion method.

### Statistical data analysis

All statistical analysis was conducted using GraphPad PRISM version 8.4.3 (GraphPad, USA). To assess discrepancies between various groups and determine the statistical significance of these variations, the One-Way or two-way analysis of variance (ANOVA) was employed, with a pre-established significance level of 0.05 (*p*-value < 0.0 5). All graphical representations of data indicate mean ± standard deviation (SD).

## Results

### The successful overexpression of miR-107 Enhances CHO cell growth and viability

The successful transfection of miR-107 was verified through fluorescence microscopy, which revealed that the transfected CHO-K1 cells displayed a distinct green fluorescence signal (Fig. 1A). This was further supported by qPCR analysis, which demonstrated a significant increase in miR-107 expression, approximately 400-fold, in cells subjected to miR-107 transfection compared to Mock and NT groups (Fig. 1C; p < 0.0001).

**Fig. 2:**
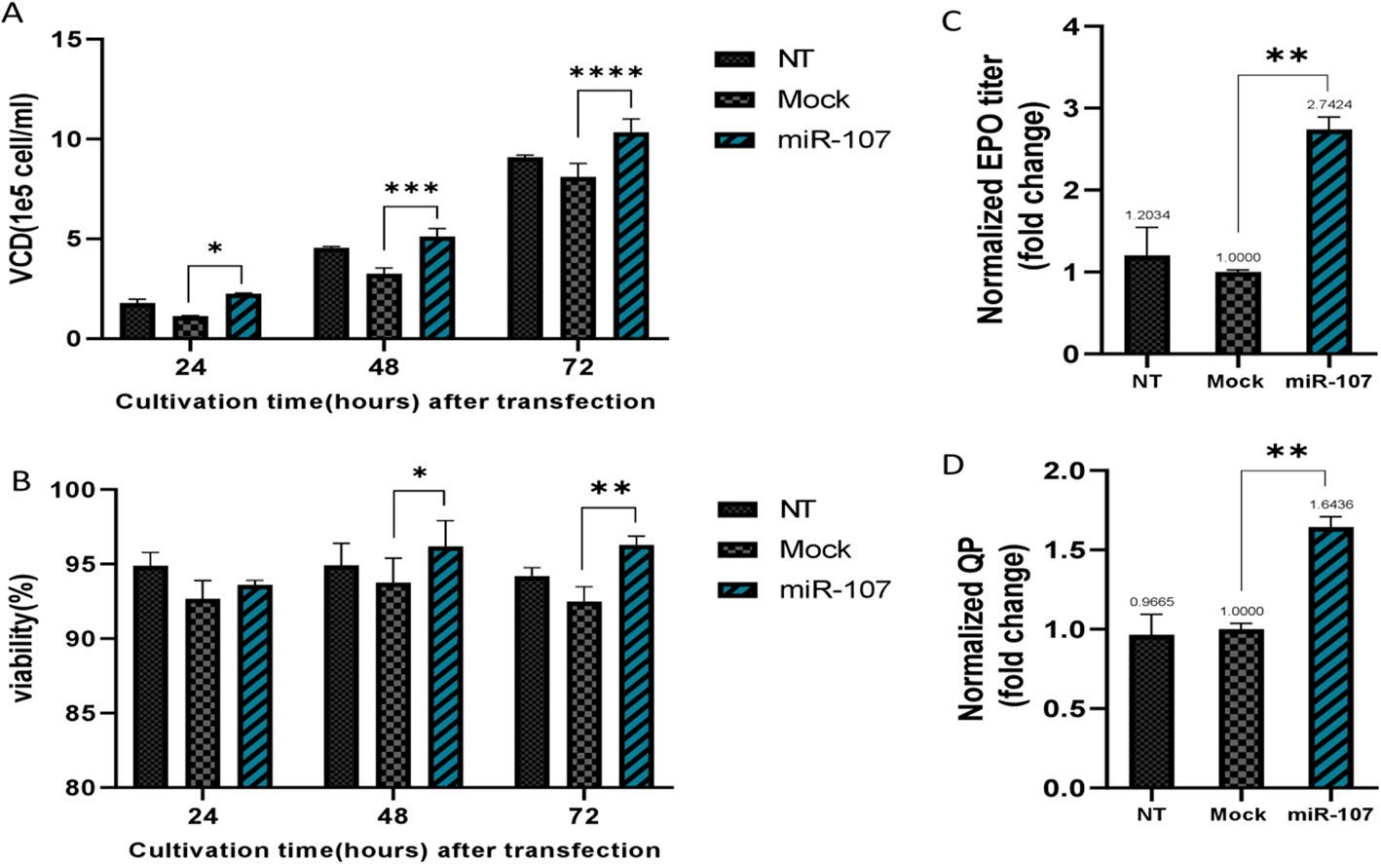
Comparative analysis of cell growth, viability, EPO titer, and specific cell productivity (q_p_). **A**, **B** Effect of miR-107 overexpression on viable cell density and cell viability of CHO-hEPO cells during a 3-day culture period after transfection, respectively. **C** Comparative analysis of total EPO titer in miR-107 transfected CHO cells with NT and Mock controls, showing a 2.7-fold increase in miR-107 overexpressed cells productivity. **D** Comparative analysis of specific cell productivity (q_p_) in miR-107 transfected CHO cells than NT and Mock controls, revealing a 1.6-fold increase in q_p_ of miR-107 overexpressed cells (* *p* < 0.05, ** p < 0.01, *** p < 0.001, **** p < 0.001)

Cell proliferation and viability are essential factors that exert a substantial influence on total productivity. A comparative assessment of growth patterns across cell groups was conducted. Figure 2A showcases an evident increase in peak VCD in CHO-hEPO cells expressing miR-107 compared to Mock but showed a slight increase compared to NT (p < 0.05). Notably, the growth of Mock appeared slightly decreased compared to NT, possibly attributed to transfection-induced stress. Also, the viability of both miR-107 expressing CHO cells and Mock on the day following transfection exhibited a modest decline, potentially attributable to the transfection reagent’s transient toxicity. However, within 48 and 72 h post-transfection, cells overexpressing miR-107 demonstrated a significant recovery in viability compared to both Mock and NT cells (*p* < 0.05) (Fig. 2B).

### Overexpression of miR-107 promotes cell cycle progression

**Fig. 3:**
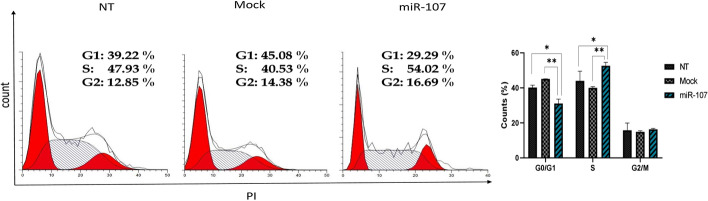
miR-107 promotes cell cycle progression. Cell cycle analysis of CHO cells was conducted using flow cytometry. The results revealed enhanced cell cycle progression due to miR-107 overexpression compared to Mock cells. Fractions of cell cycle phases were compared using a two-way analysis of variance (ANOVA). * p < 0.05, ** p < 0.01

Given that cell proliferation is closely associated with the progression of the cell cycle, we conducted a comparative analysis of the cell-cycle profiles between cells overexpressing miR-107 and control cells. In the NT cell group, approximately 40.19% ± 1.36 standard deviation (SD) of the cells was observed to be in the G0/G1 phase, while 44.03% ± 5.52 SD were found in the S phase, and 15.79% ± 4.16 SD were in the G2/M phase. Comparatively, in the Mock group, 45.11% ± 0.04 SD of the cells were in the G0/G1 phase, 40.03% ± 0.71 SD were found in the S phase, and 14.86% ± 0.67 SD were in the G2/M phase. In the miR-107 group, 31.06% ± 2.5 of the cells were in the G0/G1 phase, 52.63% ± 1.96 SD were found in the S phase, and 16.31% ± 0.54 SD were in the G2/M phase. The analysis revealed that the overexpression of miR-107 led to a reduction in the proportion of cells residing in the G1/G0 phase (0.66-fold) while concurrently increasing the accumulation of cells in the S phase (1.45-fold) in comparison with the control group (p < 0.01; Fig. 3). These findings suggest that miR-107 orchestrates its regulatory influence on cell growth by fostering cell cycle progression, specifically at the G1/S transition phase. These findings align with the observed increase in cell proliferation in miR-107 overexpressing cells. Therefore, it could be argued that the observed acceleration in cellular growth exhibited by cells over-expressing miR-107 can be attributed to an augmented proportion of cells in the S phase.

### The Overexpression of miR-107 led to a Significant Enhancement in both the Specific Productivity and EPO Titer

We conducted a study to investigate the impact of miR-107 overexpression in CHO-hEPO cells on the production of specific productivity and final EPO titer. To address this inquiry, we collected the cell culture supernatant from cells transfected with miR-107, as well as from Mock and NT cells. Subsequently, we measured the concentration of secreted EPO. The EPO titer was quantified at 1.49 µg/ml ± 0.4 SD for the NT group, 1.23 µg/ml ± 0.3 SD for the mock group, and 3.4 µg/ml ± 0.18 SD for the miR-107 group. The measurement of qp resulted in values of 2.3 pg/cell/day (pcd) ± 0.3 SD for the NT group, 2.4 pcd ± 0.09 SD for the mock group, and 4 pcd ± 0.16 SD for the miR-107 group. As shown in Fig. 2 (C, D), miR-107 overexpressing cells exhibited a remarkable 2.7-fold increase in EPO titer and a significant 1.6-fold increase in specific productivity when compared to the control cells (p < 0.01). Hence, it appears that the overexpression of miR-107 not only enhances cellular proliferation but also influences protein production in CHO-hEPO cells.

### Potential molecular insights into miR-107-mediated pathways

Evidence suggests that miR-107 holds the potential to emerge as a noteworthy contender for promoting cell growth and augmenting protein production. As depicted in Additional file 2: Figure S3, miR-107 interfaces with an array of cellular processes, including promoting proliferation and protein synthesis, alongside inhibiting ubiquitin-mediated proteolysis and apoptosis.

miR-107 has been shown to contribute to the regulation of cell cycle progression and the promotion of tumor cell survival by modulating various intracellular signaling pathways. Numerous investigations on cancer have highlighted the oncogenic effects of miR-107. It has been proven that miR-107 exerts its influence on cell growth by selectively targeting and regulating various proteins that play a crucial role in cell proliferation, such as FOXO1, TGFBR2, LET-7, DAPK, KLF4, PTEN, BTRC, LATS2, TDG, CASPASE-1, GSDMD-N, TLR4, TPM1, WNT3A, PAR4 (PAWR), CDK8, NF1, PLD1/2, PKC, FAT4, AXIN2, SIAH1, CD36, TRAF3 and DIABLO (SMAC) (Chen et al. 2012, 2011, 2019; Gao et al. 2019; Han et al. 2020, 2022; Jiang et al. 2017; Jin et al. 2022; Li et al. 2020, 2014, 2018, 2021; Liu et al. 2020; Liu and Xie 2018; Pinho et al. 2020; Qian et al. 2021; Ren et al. 2019; Roldo et al. 2006; Shrestha et al. 2014; Song et al. 2014; Wang et al. 2019, 2018, 2016; Zhang et al. 2015a, b, 2019a, 2015a; Zhang et al. 2020, 2016; Zhao et al. 2019).

Additionally, the bioinformatics algorithm has identified several predicted genes, including BTRC, FOXO, PTEN, LATS2, NF1, TGFBR2, and AXIN2, that overlap with validated genes. From the identified target genes, the *LATS2* and *PTEN* genes were selected because of their significant involvement in proliferation and protein synthesis pathways. We compared the expression levels of *LATS2* and *PTEN*, as well as their downstream genes (*TSC1, mTOR1, S6K, YAP,* and *MYC*), in cells expressing miR-107 and in control cells.

**Fig. 4:**
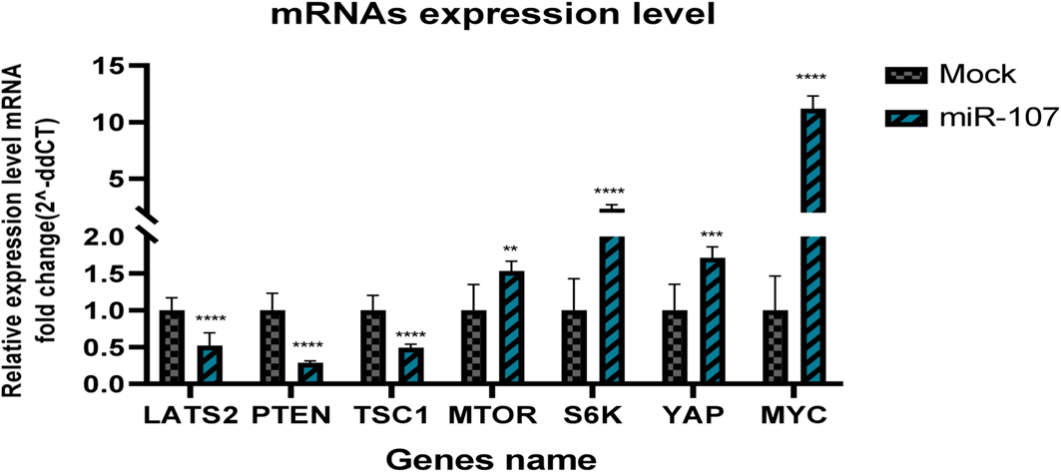
Comparative analysis of mRNA expression levels. The mRNA expression levels of *LATS2, PTEN, TSC1, MYC, YAP, MTOR,* and *S6K* using qRT-PCR in miR-107 transfected cells were compared to cells transfected with PB513b as a Mock control cell. The mRNA expression levels are illustrated as fold-change (2 ^−ddct^) relative to the controls and normalized to beta-actin. The significance of differences between miR-107 transfected cells and controls was verified (* *p* < 0.05, ** p < 0.01, *** p < 0.001, **** p < 0.001)

The qPCR analysis indicated that miR-107 expressing cells significantly reduced *LATS2, PTEN,* and *TSC1* gene expression by approximately 2, 3.5, and 2 times, respectively, compared to the control cells (Fig. 4; *p* < 0.01). Furthermore, these data revealed substantial elevations in the expression levels of *YAP, mTOR, S6K,* and *MYC* genes, with increases of approximately 1.7, 1.5, 2.3, and 11 times, respectively, in cells overexpressing miR-107 compared to control cells (*p* < 0.01). These findings suggested that miR-107 plays a pivotal role in modulating these crucial genes, contributing to enhanced cell growth and protein production.

Based on the acquired findings, we have presented a theoretical framework elucidating the potential mechanism of miR-107's functionality. By repressing *PTEN* and activating the PI3K pathway, miR-107 enhances protein synthesis through mTOR1 stimulation. Notably, miR-107 leads to elevated *YAP* levels by suppressing *LATS2*. Consequently, YAP activation triggers the *MYC* and mTOR1 pathways (Wang et al. 2021)), which leads to increased proliferation and protein synthesis (Additional file 2: Figure S4).

## Discussion

Chinese Hamster Ovary (CHO) cells have become a cornerstone in producing complex and challenging proteins and glycosylated proteins (Keysberg et al. 2021). Various genetic engineering strategies have been employed to boost productivity. In recent years, miRNAs have emerged as promising tools for enhancing CHO cell productivity by modulating cellular mechanisms such as proliferation, apoptosis, and post-translational modification (Inwood et al. 2018; Jadhav et al. 2013). The manipulation of miRNAs in CHO cells to enhance productivity has been a focal point of numerous investigations (Tihanyi and Nyitray 2020).

The oncogenic characteristics of miR-107 have been observed across diverse cancer studies. miR‐107 is known to regulate multiple intracellular signaling mediators, contributing to cell cycle progression and bolstering tumor cell survival. Extensive research has been dedicated to comprehending the impact of miR-107 on cell growth and proliferation across various human cancers, as described in the previous section.

Notably, an investigation on ovarian cancer revealed that miR-107-mimic significantly increased cell proliferation and invasiveness in SKOV3 cells. This study also showed that miR-107 can modulate the XIAP/caspase-3 signaling pathway by targeting SMAC (Han et al. 2022). Furthermore, elevated expression of miR-107 has been noted in bladder cancer (Yu et al. 2018), breast cancer (Pan et al. 2023), and gastric cancer (Chen et al. 2019). In these contexts, miR-107 directly targets PTEN, facilitating the activation of the PI3K/AKT signaling pathways. This assertion is corroborated by the consistent inverse relationship between miR-107 levels and PTEN in tumor samples (Chen et al. 2019; Pan et al. 2023; Yu et al. 2018).

However, it is noteworthy that certain studies in the field of cancer have reported findings that indicate an anti-tumor effect of miR-107. For example, elevating miR-107 levels in MiaPACA-2 and PANC-1 cells led to reduced cell growth in vitro, linked to the suppression of cyclin-dependent kinase 6 (CDK6), a potential target of miR-107 in pancreatic cancer (Lee et al. 2009). Another research highlighted that miR-107 suppresses the growth of prostate cancer cells through its interaction with cyclin E1 (Zhang et al. 2019a, b). The effects of miRNAs exhibit a degree of variability depending on the cell type, suggesting a context-dependent nature of their actions.

As previously stated, the selection of miR-107 was based on its oncogenic characteristics in different types of cancers and its involvement in multiple cellular pathways, such as cell proliferation, enhanced protein synthesis, and suppression of protein degradation. Consistent with our expectations, the overexpression of miR-107 in CHO-hEPO cells resulted in notable impacts on cell viability, growth, and specific productivity. Remarkably, the miR-107 overexpression led to a substantial elevation in EPO concentration by approximately 2.7-fold compared to the Mock cells. Furthermore, the growth and viability of miR-107-overexpressing cells exhibited enhancements compared to both the Mock and non-transfected control cells. Specific productivity also experienced a notable boost, showing a 1.6-fold improvement compared to the Mock and non-transfected cells.

Interestingly, the Mock cells displayed similarities with non-transfected cells regarding specific productivity. However, a slight decrease was noted in viability and viable cell density compared to non-transfected cells. This decrease could be attributed to the additional burden on the cells during transfection. Moreover, the upregulation of miR-107 significantly promoted cell proliferation, as evidenced by a higher proportion of cells in the S phase of the cell cycle. This finding indicates a direct correlation between miR-107 overexpression and cell cycle progression.

The qPCR analysis revealed a 400-fold increase in miRNA expression in cells transfected with miR-107. This heightened expression of miR-107 in our CHO-hEPO cells, coupled with its recognized role in regulating cell proliferation, suggests that the overexpression of miR-107 contributes to the increased VCD and specific productivity of CHO cells. The miR-107 effects on CHO-hEPO cells could be attributed to the downregulation of *LATS2, PTEN*, and *TSC1* genes, as well as the upregulation of *mTOR, S6K, MYC,* and *YAP*.

The PTEN (phosphatase and tensin homolog) protein, which functions as a tumor suppressor, counteracts the effects of the PI3K pathway. This antagonist role extends to PI3K, an upstream activator of the mammalian Target of Rapamycin (mTOR). By suppressing PTEN and activating the PI3K pathway, protein synthesis could be enhanced through mTOR1 stimulation(Song et al. 2012). The mTOR emerges as a central orchestrator among the key players in regulating protein synthesis. Its far-reaching influence spans transcription, translation, and protein synthesis, culminating in the regulation of cell growth (Asnaghi et al. 2004). The inhibition of mTOR is relieved by the downregulation of the *TSC1* gene, which acts as an inhibitor of mTOR. As part of this intricate network, the Ribosomal Protein S6 Kinase B1 (*S6K*) gene plays a pivotal role. This gene fuels protein synthesis, cellular growth, and proliferation within the mTOR signaling pathway.

The Large Tumor Suppressor Kinase 2 (*LATS2*) gene regulates the Yes-Associated Protein (YAP). The downregulation of *LATS2* expression results in elevated *YAP* levels, a vital factor in cell growth and proliferation (Croci et al. 2017; Goodman et al. 2015). On another front, *MYC* is classified as a proto-oncogene encoding a nuclear phosphoprotein that actively participates in various cellular processes, including apoptosis and cell cycle progression. Amplification of the *MYC* gene is a common occurrence in different types of human cancers (Chen et al. 2018; Chen and Olopade 2008; Kalkat et al. 2017; Schaafsma et al. 2021).

It seems that the downregulation of *PTEN* and *TSC1* can potentially activate the PI3K pathway, subsequently resulting in the upregulation of mTOR and S6K, thereby enhancing protein synthesis. Additionally, the increase in *YAP* levels by suppressing *LATS2* further promotes cell proliferation and protein synthesis by activating *MYC* and *mTOR1* (refer to Additional file 2: Figure S4).

Moreover, there is potential for future investigation into the remaining target genes, such as BTRC, FOXO1, NF1, TGFBR2, and AXIN2. BTRC is involved in various pathways, including the regulation of activated PAK-2p34 through proteasome-mediated degradation (Toma-Fukai and Shimizu 2021; Winston et al. 1999; Zhou et al. 2013). FOXO1 plays a pivotal role in various essential cellular processes by regulating gene expression programs that govern apoptosis, cell-cycle progression, and resistance to oxidative stress (Carter and Brunet 2007). NF1 functions as a tumor suppressor gene. This particular gene serves as a GTPase-activating protein, which plays a crucial role in inhibiting the activity of the RAS/MAPK pathway by expediting the hydrolysis of GTP bound to Ras(Upadhyaya et al. 2012). TGFBR2 regulates the transcription of genes associated with cell proliferation, cell cycle arrest, and tumorigenesis (Sivadas and Kannan 2014; Wieser et al. 1995). The AXIN2 gene plays a crucial role in regulating the Wnt/β-catenin signaling pathway. This pathway involves various cellular processes such as cell proliferation, migration, apoptosis, and other functions (Li et al. 2015). A more comprehensive analysis of the expression levels of these potential target genes could provide valuable insights into the intricate mechanism exerted by miR-107.

Multiple efforts have been made to enhance the expression efficiency of challenging proteins in CHO cells through miRNA manipulations. Notably, XU et al. investigated the impact of miR-106b overexpression on IgG-producing CHO cells, reporting a substantial increase of 66% in the volumetric productivity of CHO cells (Xu et al. 2019b). Additionally, Strotbek and colleagues indicated the beneficial effects of stable miR557-miR1287 overexpression in IgG1-expressing CHO cells, improving viable cell density and specific productivity (Strotbek et al. 2013). A recent study focused on miR-7, utilizing sponge decoy technology for stable miRNA depletion. This investigation revealed a 65% increase in cell growth, enhanced viability, and an impressive over threefold boost in the yield of secreted IgG protein in CHO-K1 cells (Coleman et al. 2019). Recently, the overexpression of miR-32 in CHO cells led to a notable enhancement in growth rate and a remarkable 1.8-fold increase in productivity (Bazaz et al. 2023).

In conclusion, this research represents the inaugural endeavor to explore the impact of miR-107 overexpression on CHO cell productivity within our current knowledge scope. As widely acknowledged, the volumetric titer of the protein is directly proportional to both the viable cell density (VCD) and the specific productivity (q_p_). Our research findings demonstrated that introducing miR-107 into CHO-hEPO cells engenders elevated VCD and q_p_. To conduct more extensive research, it is advisable to employ diverse strains or varieties of CHO cells, as well as alternative industrial host cells like HEK or SP2/0. Also, it is recommended to utilize different protein-producing cells, such as ETN or IgG-producing cells. Additionally, conducting proteomics analysis is recommended to gain deeper insights into the underlying mechanisms involving miR-107. Furthermore, the synergistic combination of this miRNA with others possessing regulatory roles in growth and productivity could present promising avenues for CHO cell engineering and subsequent productivity enhancement.

### Supplementary Information

**Additional file 1. ** Sheet 1: miR-107’s target genes predicted by 4 databases (miRWalk, miRanda, RNA22 and Targetscan). Sheet 2: miR-107’s target genes identified by MiRTarBase. Sheet 3: miR-107’s target genes identified through a literature review.

**Additional file 2.** **Table S1.** Sequences of primers and stem loops.** Figure S1.** Conserved sequences of mir-107 in human, mouse, and Chinese hamster.** Figure S2.** The cassette harboring miR-107: The flanking regions are depicted in green, the loop region is represented by the purple sequence, the miR-107 sequence is denoted by the red sequence, and the miR-107* (antisense) sequence is indicated by the orange region.** Figure S3.** miR-107 target gene pathways. The schematic representation illustrates pathways involving miR-107 target genes. The color-coded shapes provide insight into the regulatory impact of miR-107 on gene expression. Green shapes indicate downregulated genes, while purple shapes signify upregulated genes. The lozenge shapes correspond to validated target genes of miR-107, identified through literature review and MiRTarBase. The parallelogram shapes denote predicted target genes of miR-107, detected using predictive algorithms. Additionally, circle shapes represent target genes of miR-107 identified through a combination of validated and predicted approaches.** Figure S4.** Schematic view of our theoretical framework for the mechanism of miR-107 in CHO cells. The data obtained in qPCR analysis revealed the downregulation of LATS2, PTEN and TSC1 genes, as indicated by green arrows pointing downwards. At the same time, the upregulation of YAP, MYC, mTOR, and S6K were denoted by purple arrows pointing upwards.

## Data Availability

The data utilized in this study will be accessible upon reasonable request.

## Abbreviations

CHO: Chinese hamster ovary
miR-107: MicroRNA-107
MiRNAs: MicroRNAs
FBS: Fetal Bovine Serum
GFP: Green fluorescent Protein
VCD: Viable cell density
qp: Specific productivity
pcd: Picogram/cell/day
EPO: Erythropoietin
PI3K: Phosphatidylinositol-3 kinases
PTEN: Phosphatase and tensin homolog
TSC1: Tuberous Sclerosis 1
LATS2: Large Tumor Suppressor Kinase 2
mTOR: Mammalian target of rapamycin
S6K: Ribosomal Protein S6 Kinase B1
YAP: Yes-Associated Protein
BTRC: Beta-Transducin Repeat Containing E3 Ubiquitin Protein Ligase
FOXO1: Forkhead Box O1
NF1: Neurofibromin 1
TGFBR2: Transforming Growth Factor Beta Receptor 2

## References

[CR1] Asnaghi L, Bruno P, Priulla M, Nicolin A (2004). mTOR: a protein kinase switching between life and death. Pharmacol Res.

[CR2] Bartel DP (2009). MicroRNAs: target recognition and regulatory functions. Cell.

[CR3] Bazaz M, Adeli A, Azizi M, Karimipoor M, Mahboudi F, Davoudi N (2023). Overexpression of miR-32 in Chinese hamster ovary cells increases production of Fc-fusion protein. AMB Express.

[CR4] Berezikov E (2011). Evolution of microRNA diversity and regulation in animals. Nat Rev Genet.

[CR5] Brinkrolf K, Rupp O, Laux H, Kollin F, Ernst W, Linke B, Kofler R, Romand S, Hesse F, Budach WE (2013). Chinese hamster genome sequenced from sorted chromosomes. Nat Biotechnol.

[CR6] Carter ME, Brunet A (2007). FOXO transcription factors. Curr Biol.

[CR7] Chen Y, Olopade OI (2008). MYC in breast tumor progression. Expert Rev Anticancer Ther.

[CR8] Chen P-S, Su J-L, Cha S-T, Tarn W-Y, Wang M-Y, Hsu H-C, Lin M-T, Chu C-Y, Hua K-T, Chen C-N (2011). miR-107 promotes tumor progression by targeting the let-7 microRNA in mice and humans. J Clin Investig.

[CR9] Chen H-Y, Lin Y-M, Chung H-C, Lang Y-D, Lin C-J, Huang J, Wang W-C, Lin F-M, Chen Z, Huang H-D (2012). miR-103/107 promote metastasis of colorectal cancer by targeting the metastasis suppressors DAPK and KLF4miR-103/107 Target DAPK/KLF4 in CRC Metastasis. Can Res.

[CR10] Chen H, Liu H, Qing G (2018). Targeting oncogenic Myc as a strategy for cancer treatment. Signal Transduct Target Ther.

[CR11] Chen P, Zhao X, Wang H, Zheng M, Wang Q, Chang W (2019). The down-regulation of lncRNA PCAT18 promotes the progression of gastric cancer via MiR-107/PTEN/PI3K/AKT signaling pathway. OncoTargets Therapy.

[CR12] Coleman O, Suda S, Meiller J, Henry M, Riedl M, Barron N, Clynes M, Meleady P (2019). Increased growth rate and productivity following stable depletion of miR-7 in a mAb producing CHO cell line causes an increase in proteins associated with the Akt pathway and ribosome biogenesis. J Proteomics.

[CR13] Croci O, De Fazio S, Biagioni F, Donato E, Caganova M, Curti L, Doni M, Sberna S, Aldeghi D, Biancotto C (2017). Transcriptional integration of mitogenic and mechanical signals by Myc and YAP. Genes Development.

[CR14] Dahodwala H, Lee KH (2019). The fickle CHO: a review of the causes, implications, and potential alleviation of the CHO cell line instability problem. Curr Opin Biotechnol.

[CR15] Fischer S, Handrick R, Otte K (2015). The art of CHO cell engineering: A comprehensive retrospect and future perspectives. Biotechnol Adv.

[CR16] Fischer S, Paul AJ, Wagner A, Mathias S, Geiss M, Schandock F, Domnowski M, Zimmermann J, Handrick R, Hesse F (2015). miR-2861 as novel HDAC5 inhibitor in CHO cells enhances productivity while maintaining product quality. Biotechnol Bioeng.

[CR17] Gao Y-W, Ma F, Xie Y-C, Ding M-G, Luo L-H, Jiang S, Rao L, Liu X-L (2019). Sp1-induced upregulation of the long noncoding RNA TINCR inhibits cell migration and invasion by regulating miR-107/miR-1286 in lung adenocarcinoma. Am J Transl Res.

[CR18] Goodman CA, Dietz JM, Jacobs BL, McNally RM, You J-S, Hornberger TA (2015). Yes-Associated Protein is up-regulated by mechanical overload and is sufficient to induce skeletal muscle hypertrophy. FEBS Lett.

[CR19] Hackl M, Jakobi T, Blom J, Doppmeier D, Brinkrolf K, Szczepanowski R, Bernhart SH, Zu Siederdissen CH, Bort JAH, Wieser M (2011). Next-generation sequencing of the Chinese hamster ovary microRNA transcriptome: Identification, annotation and profiling of microRNAs as targets for cellular engineering. J Biotechnol.

[CR20] Han H, Li H, Zhou J (2020). Long non-coding RNA MIR503HG inhibits the proliferation, migration and invasion of colon cancer cells via miR-107/Par4 axis. Exp Cell Res.

[CR21] Han Z, Li D, Yang Y, Zhang H (2022). LINC-DUBR suppresses malignant progression of ovarian cancer by downregulating miR-107 to induce SMAC expression. J Healthcare Eng.

[CR22] Huhn S, Ou Y, Kumar A, Liu R, Du Z (2019). High throughput, efficacious gene editing & genome surveillance in Chinese hamster ovary cells. PLoS ONE.

[CR23] Inwood S, Betenbaugh MJ, Shiloach J (2018). Methods for using small non-coding RNAs to improve recombinant protein expression in mammalian cells. Genes.

[CR24] Jadhav V, Hackl M, Druz A, Shridhar S, Chung C-Y, Heffner KM, Kreil DP, Betenbaugh M, Shiloach J, Barron N (2013). CHO microRNA engineering is growing up: recent successes and future challenges. Biotechnol Adv.

[CR25] Jadhav V, Hackl M, Klanert G, Bort JAH, Kunert R, Grillari J, Borth N (2014). Stable overexpression of miR-17 enhances recombinant protein production of CHO cells. J Biotechnol.

[CR26] Jelkmann W (2013). Physiology and pharmacology of erythropoietin. Transfusion Med Hemotherapy.

[CR27] Jelkmann W (2016). Erythropoietin. Sports Endocrinol.

[CR28] Jiang R, Zhang C, Liu G, Gu R, Wu H (2017). MicroRNA-107 promotes proliferation, migration, and invasion of osteosarcoma cells by targeting tropomyosin 1. Oncol Res Feat Preclin Clin Cancer Ther.

[CR29] Jin D, Guo J, Wu Y, Yang L, Wang X, Du J, Dai J, Chen W, Gong K, Miao S (2022). Correction: m6A demethylase ALKBH5 inhibits tumor growth and metastasis by reducing YTHDFs-mediated YAP expression and inhibiting miR-107/LATS2–mediated YAP activity in NSCLC. Mol Cancer.

[CR30] Kalkat M, De Melo J, Hickman KA, Lourenco C, Redel C, Resetca D, Tamachi A, Tu WB, Penn LZ (2017). MYC deregulation in primary human cancers. Genes.

[CR31] Kaneyoshi K, Kuroda K, Uchiyama K, Onitsuka M, Yamano-Adachi N, Koga Y, Omasa T (2019). Secretion analysis of intracellular “difficult-to-express” immunoglobulin G (IgG) in Chinese hamster ovary (CHO) cells. Cytotechnology.

[CR32] Kelly PS, Breen L, Gallagher C, Kelly S, Henry M, Lao NT, Meleady P, O'Gorman D, Clynes M, Barron N (2015). Re-programming CHO cell metabolism using miR-23 tips the balance towards a highly productive phenotype. Biotechnol J.

[CR33] Keysberg C, Hertel O, Schelletter L, Busche T, Sochart C, Kalinowski J, Hoffrogge R, Otte K, Noll T (2021). Exploring the molecular content of CHO exosomes during bioprocessing. Appl Microbiol Biotechnol.

[CR34] Kim JY, Kim Y-G, Lee GM (2012). CHO cells in biotechnology for production of recombinant proteins: current state and further potential. Appl Microbiol Biotechnol.

[CR35] Kunert R, Reinhart D (2016). Advances in recombinant antibody manufacturing. Appl Microbiol Biotechnol.

[CR36] Lai T, Yang Y, Ng SK (2013). Advances in mammalian cell line development technologies for recombinant protein production. Pharmaceuticals.

[CR37] Lalonde M-E, Durocher Y (2017). Therapeutic glycoprotein production in mammalian cells. J Biotechnol.

[CR38] Lee K-H, Lotterman C, Karikari C, Omura N, Feldmann G, Habbe N, Goggins MG, Mendell JT, Maitra A (2009). Epigenetic silencing of MicroRNA miR-107 regulates cyclin-dependent kinase 6 expression in pancreatic cancer. Pancreatology.

[CR39] Lewis NE, Liu X, Li Y, Nagarajan H, Yerganian G (2013). Genomic landscapes of Chinese hamster ovary cell lines as revealed by the Cricetulus griseus draft genome. Nat Biotechnol.

[CR40] Li F, Liu B, Gao Y, Liu Y, Xu Y, Tong W, Zhang A (2014). Upregulation of microRNA-107 induces proliferation in human gastric cancer cells by targeting the transcription factor FOXO1. FEBS Lett.

[CR41] Li S, Wang C, Liu X, Hua S, Liu X (2015). The roles of AXIN2 in tumorigenesis and epigenetic regulation. Fam Cancer.

[CR42] Li H, Wei X, Yang J, Dong D, Hao D, Huang Y, Lan X, Plath M, Lei C, Ma Y (2018). circFGFR4 promotes differentiation of myoblasts via binding miR-107 to relieve its inhibition of Wnt3a. Molecular Therapy-Nucleic Acids.

[CR43] Li D, Chai L, Yu X, Song Y, Zhu X, Fan S, Jiang W, Qiao T, Tong J, Liu S (2020). The HOTAIRM1/miR-107/TDG axis regulates papillary thyroid cancer cell proliferation and invasion. Cell Death Dis.

[CR44] Li W, Lu H, Wang H, Ning X, Liu Q, Zhang H, Liu Z, Wang J, Zhao W, Gu Y (2021). Circular RNA TGFBR2 acts as a ceRNA to suppress nasopharyngeal carcinoma progression by sponging miR-107. Cancer Lett.

[CR45] Liu H, Xie H (2018). Overexpression of miR-107 promotes the proliferation and tumorigenic ability of HT29 cells. Chin J Cell Mol Immunol.

[CR46] Liu H-N, Dong W-H, Wang T (2022). The Effect of microRNA on the Production of Recombinant Protein in CHO Cells and its Mechanism. Front Bioeng Biotechnol.

[CR47] Liu F, Liu S, Ai F, Zhang D, Xiao Z, Nie X, Fu Y (2020) miR-107 Promotes Proliferation and Inhibits Apoptosis of Colon Cancer Cells by Targeting Prostate Apoptosis Response-4 (Par4). 10.3727/096504016X14803476672380 PMC7841080 27938501

[CR48] Maccani A, Hackl M, Leitner C, Steinfellner W, Graf AB, Tatto NE, Karbiener M, Scheideler M, Grillari J, Mattanovich D (2014). Identification of microRNAs specific for high producer CHO cell lines using steady-state cultivation. Appl Microbiol Biotechnol.

[CR49] Mullard A (2021). FDA approves 100th monoclonal antibody product. Nat Rev Drug Discovery.

[CR50] O’Flaherty R, Bergin A, Flampouri E, Mota LM, Obaidi I, Quigley A, Xie Y, Butler M (2020). Mammalian cell culture for production of recombinant proteins: A review of the critical steps in their biomanufacturing. Biotechnol Adv.

[CR51] Pan H, Peng H, Dai Y, Han B, Yang G, Jiang N, Zhou P (2023). Effects of miR-107 on Breast Cancer Cell Growth and Death via Regulation of the PTEN/AKT Signaling Pathway. J Oncol.

[CR52] Peng Y, Croce CM (2016). The role of MicroRNAs in human cancer. Signal Transduct Target Ther.

[CR53] Pinho JD, Silva GEB, Júnior AALT, de Castro Belfort MR, Macedo JM, da Cunha IW, Quintana LG, Calixto J (2020). MIR-107, MIR-223-3P and MIR-21-5P reveals potential biomarkers in penile cancer. Asian Pacific J Cancer Prevent.

[CR54] Qian J, Fu P, Li S, Li X, Chen Y, Lin Z (2021). miR-107 affects cartilage matrix degradation in the pathogenesis of knee osteoarthritis by regulating caspase-1. J Orthop Surg Res.

[CR55] Raab N, Mathias S, Alt K, Handrick R, Fischer S, Schmieder V, Jadhav V, Borth N, Otte K (2019). CRISPR/Cas9-mediated knockout of microRNA-744 improves antibody titer of CHO production cell lines. Biotechnol J.

[CR56] Ren W, Zhang X, Li W, Feng Q, Feng H, Tong Y, Rong H, Wang W, Zhang D, Zhang Z (2019). Exosomal miRNA-107 induces myeloid-derived suppressor cell expansion in gastric cancer. Cancer Management and Research.

[CR57] Roldo C, Missiaglia E, Hagan JP, Falconi M, Capelli P, Bersani S, Calin GA, Volinia S, Liu C-G, Scarpa A (2006). MicroRNA expression abnormalities in pancreatic endocrine and acinar tumors are associated with distinctive pathologic features and clinical behavior. J Clin Oncol.

[CR58] Schaafsma E, Zhao Y, Zhang L, Li Y, Cheng C (2021). MYC activity inference captures diverse mechanisms of aberrant MYC pathway activation in human cancers. Mol Cancer Res.

[CR59] Schoellhorn M, Fischer S, Wagner A, Handrick R, Otte K (2017). miR-143 targets MAPK7 in CHO cells and induces a hyperproductive phenotype to enhance production of difficult-to-express proteins. Biotechnol Prog.

[CR60] Shrestha S, Hsu SD, Huang WY, Huang HY, Chen W, Weng SL, Huang HD (2014). A systematic review of microRNA expression profiling studies in human gastric cancer. Cancer Med.

[CR61] Singh A, Fan Y, Cakal S, Amann T, Hansen A, Borth N, Lee GM, Kildegaard HF, Andersen M (2022). Identification of novel miRNA targets in CHO cell lines and characterization of their impact on protein N-glycosylation. Authorea Preprints..

[CR62] Sivadas VP, Kannan S (2014). TGFBR2 (Transforming Growth Factor, Beta Receptor II (70/80kDa)). Atlas Genet Cytogenet Oncol Haematol.

[CR63] Song MS, Salmena L, Pandolfi PP (2012). The functions and regulation of the PTEN tumour suppressor. Nat Rev Mol Cell Biol.

[CR64] Song Y-Q, Ma X-H, Ma G-L, Lin B, Liu C, Deng Q-J, Lv W-P (2014). MicroRNA-107 promotes proliferation of gastric cancer cells by targeting cyclin dependent kinase 8. Diagn Pathol.

[CR65] Strotbek M, Florin L, Koenitzer J, Tolstrup A, Kaufmann H, Hausser A, Olayioye MA (2013). Stable microRNA expression enhances therapeutic antibody productivity of Chinese hamster ovary cells. Metab Eng.

[CR66] Szkodny AC, Lee KH (2022). Biopharmaceutical manufacturing: Historical perspectives and future directions. Annu Rev Chem Biomol Eng.

[CR67] Tharmalingam T, Barkhordarian H, Tejeda N, Daris K, Yaghmour S, Yam P, Lu F, Goudar C, Munro T, Stevens J (2018). Characterization of phenotypic and genotypic diversity in subclones derived from a clonal cell line. Biotechnol Prog.

[CR68] Tihanyi B, Nyitray L (2020). Recent advances in CHO cell line development for recombinant protein production. Drug Discov Today Technol.

[CR69] Toma-Fukai S, Shimizu T (2021). Structural diversity of ubiquitin E3 ligase. Molecules.

[CR70] Upadhyaya M, Thompson P, Han S, Cooper DN (2012). Neurofibromatosis type 1. Mol Diagn Genetic Diseases.

[CR71] Valdés-Bango Curell R, Barron N (2018). Exploring the potential application of short non-coding RNA-based genetic circuits in Chinese hamster ovary cells. Biotechnol J.

[CR72] Wang S, Ma G, Zhu H, Lv C, Chu H, Tong N, Wu D, Qiang F, Gong W, Zhao Q (2016). miR-107 regulates tumor progression by targeting NF1 in gastric cancer. Sci Rep.

[CR73] Wang S, Kobeissi A, Dong Y, Kaplan N, Yang W, He C, Zeng K, Peng H (2018). MicroRNAs-103/107 regulate autophagy in the epidermis. J Investig Dermatol.

[CR74] Wang L, Li K, Wang C, Shi X, Yang H (2019). miR-107 regulates growth and metastasis of gastric cancer cells via activation of the PI3K-AKT signaling pathway by down-regulating FAT4. Cancer Med.

[CR75] Wang Y, Zhang Y, Feng X, Tian H, Fu X, Gu W, Wen Y (2021). Metformin inhibits mTOR and c-Myc by decreasing YAP protein expression in OSCC cells. Oncol Rep.

[CR76] Wieser R, Wrana JL, Massague J (1995). GS domain mutations that constitutively activate T beta R-I, the downstream signaling component in the TGF-beta receptor complex. EMBO J.

[CR77] Winston JT, Strack P, Beer-Romero P, Chu CY, Elledge SJ, Harper JW (1999). The SCFβ-TRCP–ubiquitin ligase complex associates specifically with phosphorylated destruction motifs in IκBα and β-catenin and stimulates IκBα ubiquitination in vitro. Genes Dev.

[CR78] Xu X, Nagarajan H, Lewis NE, Pan S, Cai Z, Liu X, Chen W, Xie M, Wang W, Hammond S (2011). The genomic sequence of the Chinese hamster ovary (CHO)-K1 cell line. Nat Biotechnol.

[CR79] Xu C, Han Q, Zhou Q, Zhang L, Wu P, Lu Y, Si Y, Ma T, Ma B, Zhang B (2019). MiR-106b promotes therapeutic antibody expression in CHO cells by targeting deubiquitinase CYLD. Appl Microbiol Biotechnol.

[CR80] Xu C, Han Q, Zhou Q, Zhang L, Wu P, Lu Y, Si Y, Ma T, Ma B, Zhang B (2019). MiR-106b promotes therapeutic antibody expression in CHO cells by targeting deubiquitinase CYLD. Appl Microbiol Biotechnol.

[CR81] Yu Q-F, Liu P, Li Z-Y, Zhang C-F, Chen S-Q, Li Z-H, Zhang G-Y, Li J-C (2018). MiR-103/107 induces tumorigenicity in bladder cancer cell by suppressing PTEN. Eur Rev Med Pharmacol Sci.

[CR82] Zhang J-J, Wang C-Y, Hua L, Yao K-H, Chen J-T, Hu J-H (2015). miR-107 promotes hepatocellular carcinoma cell proliferation by targeting Axin2. Int J Clin Exp Pathol.

[CR83] Zhang M, Wang X, Li W, Cui Y (2015). miR-107 and miR-25 simultaneously target LATS2 and regulate proliferation and invasion of gastric adenocarcinoma (GAC) cells. Biochem Biophys Res Commun.

[CR84] Zhang L, Ma P, Sun L (2016). MiR-107 down-regulates SIAH1 expression in human breast cancer cells and silencing of miR-107 inhibits tumor growth in a nude mouse model of triple-negative breast cancer. Mol Carcinog.

[CR85] Zhang X, Jin K, Luo J, Liu B, Xie L (2019). MicroRNA-107 inhibits proliferation of prostate cancer cells by targeting cyclin E1. Neoplasma.

[CR86] Zhang X, Yao J, Shi H, Gao B, Zhang L (2019). LncRNA TINCR/microRNA-107/CD36 regulates cell proliferation and apoptosis in colorectal cancer via PPAR signaling pathway based on bioinformatics analysis. Biol Chem.

[CR87] Zhang J, Sun J, Liu J, Gu D, Shi X (2020). Correlation between microRNA-107 expression level and prognosis in patients with colorectal cancer. Int J Clin Exp Pathol.

[CR88] Zhao X, Li H, Wang L (2019). MicroRNA-107 regulates autophagy and apoptosis of osteoarthritis chondrocytes by targeting TRAF3. Int Immunopharmacol.

[CR89] Zhou W, Wei W, Sun Y (2013). Genetically engineered mouse models for functional studies of SKP1-CUL1-F-box-protein (SCF) E3 ubiquitin ligases. Cell Res.

