# Supplementary material for: Enhancing protein production and growth in chinese hamster ovary cells through miR-107 overexpression
Source: AMB Express. 2024 Feb 1;14:16. doi: 10.1186/s13568-024-01670-y (PMC10834913; doi:10.1186/s13568-024-01670-y)
Supplement: Supplementary file 2 — Additional file 2. Table S1. Sequences of primers and stem loops. Figure S1. Conserved sequences of mir-107 in human, mouse, and Chinese hamster. Figure S2. The cassette harboring miR-107: The flanking regions are depicted in green, the loop region is represented by the purple sequence, the miR-107 sequence is denoted by the red sequence, and the miR-107* (antisense) sequence is indicated by the orange region. Figure S3. miR-107 target gene pathways. The schematic representation illustrates pathways involving miR-107 target genes. The color-coded shapes provide insight into the regulatory impact of miR-107 on gene expression. Green shapes indicate downregulated genes, while purple shapes signify upregulated genes. The lozenge shapes correspond to validated target genes of miR-107, identified through literature review and MiRTarBase. The parallelogram shapes denote predicted target genes of miR-107, detected using predictive algorithms. Additionally, circle shapes represent target genes of miR-107 identified through a combination of validated and predicted approaches. Figure S4. Schematic view of our theoretical framework for the mechanism of miR-107 in CHO cells. The data obtained in qPCR analysis revealed the downregulation of LATS2, PTEN and TSC1 genes, as indicated by green arrows pointing downwards. At the same time, the upregulation of YAP, MYC, mTOR, and S6K were denoted by purple arrows pointing upwards. [file 13568_2024_1670_MOESM2_ESM.docx]

Table S1: Sequences of primers and stem loops

| Gene Name | Sequences of oligonucleotides (5'3') |
| --- | --- |
| miR-107 stem-loop | GTCGTATGCAGAGCAGGGTCCGAGGTATTCGCACTGCATACGACGATAGC |
| U6 stem-loop | GTCGTATGCAGAGCAGGGTCCGAGGTATTCGCACTGCATACGACCGCTTC |
| miR-107 forward | AGACCAGCAGCATTGTACAG |
| U6 forward | CTCGCTTCGGCAGCAC |
| Universal reverse | GAGCAGGGTCCGAGGT |
| PTEN Forward | CCAGTCAGAGACGCTATGTG |
| PTEN Reverse | GACCACAAACTGAGGATTGC |
| LATS 2 Forward | CAGCTCTGTGACTGGTGGAG |
| LATS2 Reverse | CAGCGTGTTCTCCCAGTTGA |
| TSC1 Forward | ACCGTTCAGCAGATGTCACC |
| TSC1 Reverse | GGCTCCAGTGAGTAGCTTGC |
| mTOR Forward | AGGTGTGGTTTGACCGAAGA |
| mTOR Reverse | CAGGTTGGATGGGTGTCTGT |
| S6K Forward | ATCATGCTCAATCACCAAGGTC |
| S6K Reverse | AACTCCACCAATCCACAGCA |
| MYC Forward | CACGTCTCCACTCATCAGCA |
| MYC Reverse | TCGTTTCTCCTCTGGCGTTC |
| Actin-b Forward | GCCTTCCTTCCTGGGTATG |
| Actin-b Reverse | CTTGATCTTCATGGTGCTGG |


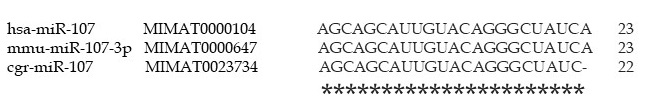
Figure S1: Conserved sequences of mir-107 in human, mouse, and Chinese hamster

**Figure S2: The cassette harboring miR-107**: The flanking regions are depicted in green, the loop region is represented by the purple sequence, the miR-107 sequence is denoted by the red sequence, and the miR-107* (antisense) sequence is indicated by the orange region.


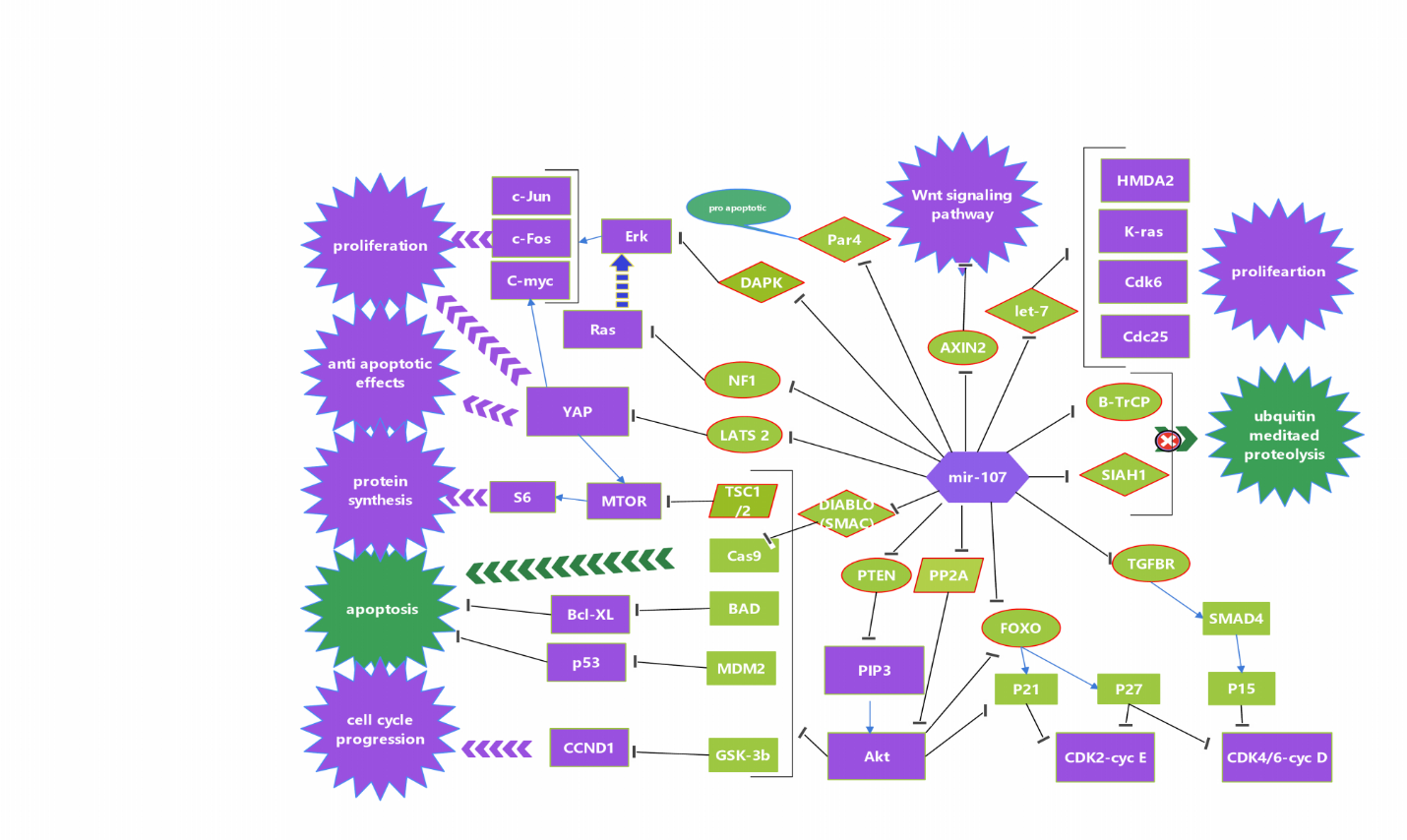


**Figure S3: miR-107 target gene pathways.** The schematic representation illustrates pathways involving miR-107 target genes. The color-coded shapes provide insight into the regulatory impact of miR-107 on gene expression. Green shapes indicate downregulated genes, while purple shapes signify upregulated genes. The lozenge shapes ( ) correspond to validated target genes of miR-107, identified through literature review and MiRTarBase. The parallelogram shapes ( ) denote predicted target genes of miR-107, detected using predictive algorithms. Additionally, circle shapes ( ) represent target genes of miR-107 identified through a combination of validated and predicted approaches.


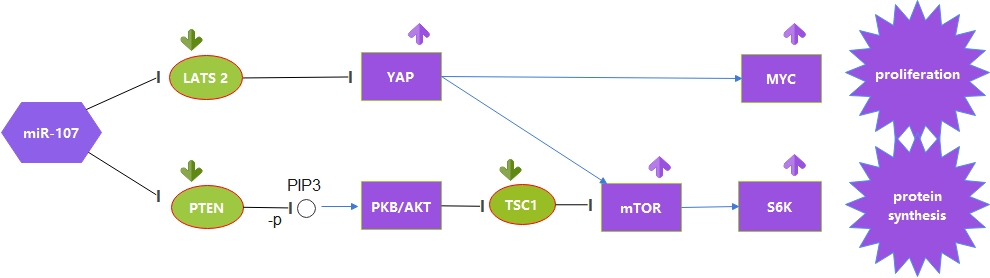


**Figure** **S4: Schematic view of our theoretical framework for the mechanism of miR-107 in CHO cells**. The data obtained in qPCR analysis revealed the downregulation of *LATS2, PTEN* and *TSC1* genes, as indicated by green arrows pointing downwards. At the same time, the upregulation of *YAP, MYC, mTOR,* and *S6K* were denoted by purple arrows pointing upwards.
